# Heartbeat Complexity Modulation in Bipolar Disorder during Daytime and Nighttime

**DOI:** 10.1038/s41598-017-18036-z

**Published:** 2017-12-20

**Authors:** Mimma Nardelli, Antonio Lanata, Gilles Bertschy, Enzo Pasquale Scilingo, Gaetano Valenza

**Affiliations:** 1 Computational Physiology and Biomedical Instruments group, Department of Information Engineering & Bioengineering and Robotics Research Centre E. Piaggio, School of Engineering, University of Pisa, Pisa, Italy; 2 Department of Psychiatry and Mental Health, Strasbourg University Hospital, INSERM U1114, University of Strasbourg - F-67000, Strasbourg, France

## Abstract

This study reports on the complexity modulation of heartbeat dynamics in patients affected by bipolar disorder. In particular, a multiscale entropy analysis was applied to the R-R interval series, that were derived from electrocardiographic (ECG) signals for a group of nineteen subjects comprised of eight patients and eleven healthy control subjects. They were monitored using a textile-based sensorized t-shirt during the day and overnight for a total of 47 diurnal and 27 nocturnal recordings. Patients showed three different mood states: depression, hypomania and euthymia. Results show a clear loss of complexity during depressive and hypomanic states as compared to euthymic and healthy control states. In addition, we observed that a more significant complexity modulation among healthy and pathological mood states occurs during the night. These findings suggest that bipolar disorder is associated with an enhanced sleep-related dysregulation of the Autonomic Nervous System (ANS) activity, and that heartbeat complex dynamics may serve as a viable marker of pathological conditions in mental health.

## Introduction

Bipolar disorder is recognized as a chronic illness with a lifetime prevalence of 1–3% and is considered one of the world’s ten most disabling conditions^1,2^. This disease is characterized by pathological mood changes, being a significant source of disquietude, suffering, and disability, often ending in suicide.

Pathological mood states in bipolar disorder include depression, mania or hypomania, mixed state, and euthymia. More specifically, depressive states are characterized by sadness, anxiety, feelings of guilt, loss of interest in activities and suicidal thoughts in some cases. Mania is characterized by a pathologically-elevated mood, with extreme happiness and irritability^3^, whereas hypomania is a less severe form of mania. During a mixed state, patients experience both manic and depressive sympthoms at the same time. The euthymic state is characterized by a normal affective balance^2^.

Given the high cost of treatment and repercussions for patients, relatives and caregivers, bipolar disorder is perceived as a major social problem^4^. Despite its prevalence and high cost of treatment^5^ (also due to the high number of mis-diagnosis and additional indirect costs, e.g., those due to work loss^6^), diagnosis of bipolar disorder is still ill-defined. Likewise, for the great majority of mental disorders, diagnosis relies on the clinician’s expertise and background, supported only by scores gathered from psychometric scales and structured interviews^2^. Furthermore, patients with mood disorders might experience a very heterogeneous pattern of symptoms related to the phenomenology, severity, number, and duration of the symptomatic episodes, as well as the time interval between them.

The diagnosis of bipolar disorder is based on clinical observation of a patient’s behavioral mood episodes, according to standardized criteria described in the fifth edition of the Diagnostic and Statistical Manual of Mental Disorders (DSM-V)^7^ and the tenth edition of the International Classification of Diseases (ICD-10)^8^. These criteria differentiate the diagnosis according to the presence, sequence and history of critical mood episodes. According to DSM-V classification, the diagnosis of depressive episodes is made if the patient exhibits five out of nine possible symptoms. In line with this approach, a patient who has had only four symptoms of depressive episodes is considered remitted, although partially remitted. Similar cutoffs are applied for the diagnosis of other types of mood episodes and clearly can lead to biased interpretation and inconsistency. Given the complexity of the clinical presentation of mood disorders, no laboratory or neuroimaging test is currently available to diagnose the disease^9^. Also due to possible comorbid somatic diseases, such as cardiovascular diseases^10–12^, obesity^13^, metabolic syndromes, hyperlipidemia, hypertension and diabetes^14^, so far neither biological markers nor physiological correlates have been found to be specific and sensitive enough for current clinical practice^15^.

Several research attempts have been recently made to try to overcome this important limitation. They consider bipolar disorder as a multi-system disorder, which involve the brain and body^16^. Previous studies have highlighted significant changes associated with mental disorders and Autonomic Nervous System (ANS) activity and, more specifically, on Heart Rate Variability (HRV)^17–23^ when compared with a healthy control group.

It is worthwhile noting that HRV represents the beat-to-beat variation of the RR intervals around their mean^17^ and can be considered as the output of a nonlinear system, given the multiple ANS signaling occurring at the level of the sinoatrial node for cardiovascular control^24^. Consequently, the quantification of heartbeat complex dynamics has been proven to provide relevant information on psychophysiological and pathological states^25^ and their variation according to external stimuli, aging and the presence of disease^25–29^. Usually, a high complexity in heartbeat dynamics is associated with a healthy cardiovascular system, underlying long-range correlations which follow fractal properties, whereas a reduced heartbeat complexity often reflects some anti-homeostatic behaviours^30–32^.

Recent evidence suggests that such a quantification of HRV irregularity should be performed at different timescales in order to estimate complexity^33–35^. A multi-scale approach for the quantification of signal regularity was first introduced by Zhang^36^, and its application to cardiovascular systems by Costa *et al*.^33,35^. Traditional entropy algorithms, such as Approximate Entropy (ApEn) and Sample Entropy (SampEn), may assign a higher value of entropy to time series during certain pathological conditions, for instance atrial fibrillation^35^, that are presumed to represent less complex dynamics^30^. A possible reason for this may be the fact that these measures are based on a single scale and biased by the specific physiological noise underlying the phenomena of interest. To this extent, MultiScale Entropy (MSE), a technique derived from SampEn analysis^37^, produces a value that reflects the mean rate of information at each level of resolution. MSE algorithm has been successfully applied in several medical applications^38–41^ including mental disorders^28,42^.

We have been particularly inspired by a study showing a significant increase of HRV regularity in patients with major depression with respect to the healthy controls, as quantified through MSE indices on data gathered from nighttime recordings^43^. Therefore, in a preliminary study we tested the hypothesis of having such a heartbeat complexity modulation also among pathological mood states associated with bipolar disorder^28^. The present study improved on MSE-related methodological issues as well as demonstrated that depressive state in bipolar disorder is associated with a higher irregularity level than hypomania, and a lower irregularity level than euthymia^28^.

Here we extend this hypothesis to patients including data gathered from daytime recordings as well as data gathered from healthy subjects undergoing daytime and nighttime recordings. This allowed us to study patterns of heartbeat complexity in bipolar disorder during the day with respect to night, as well as to investigate whether the euthymic state is associated with a lower heartbeat irregularity, rather than a healthy state. Long-term cardiovascular monitoring was performed using a comfortable, textile-based wearable system developed in the framework of European project PSYCHE (Personalised monitoring SYstems for Care in mental HEalth), where patients and healthy volunteers did not have to follow a specific experimental procedure^27,29^.

Methodological details related to signal processing and experimental setup as well as experimental results, conclusion and discussion follow below.

## Methods

### Experimental protocol

The experimental protocol implemented in this study was developed as a part of the European project PSYCHE. The acquisition system and details of the experimental procedure can be found in^27–29^.

The study was conducted at the University Hospital of Strasbourg. Daytime acquisitions were performed from 8 am to 8 pm, whereas nighttime acquisitions were performed from 8 pm to 7.30 am. All subjects were enrolled and signed the informed consent the day before data recording. All recordings were performed over a 12-hour period because of the battery autonomy of the recording device, known here as the PSYCHE platform^27–29^.

This monitoring platform consists of a comfortable, textile-based sensorized t-shirt embedded with electrodes developed by Smartex s.r.l., which is able to acquire electrocardiogram (ECG) with a sampling rate of 250 Hz. Data acquisition occurred entirely in the electronics of the wearable platform, where data were conditioned and digitalized. ECG signals were pre-filtered through a tenth order band-pass finite impulse response filter with cut-off frequencies of 0.05–35 Hz, approximated by the Butterworth polynomial. Textile electrodes could lose contact with the patient body because of movement, therefore an algorithm for automatic movement artifact removal was applied to the recorded data as reported in^27^. Then, an R-peak detection procedure was carried out according to the Pan-Tompkins algorithm^44^.

Bipolar subjects were recruited in the out-patient University clinic of Strasbourg according to the following traits as defined in the same project PSYCHE:

- age between 18 and 65;
- presenting bipolar or cyclothymic disorders according to the Diagnostic and Statistical Manual - 4th edition (DSM-IV TR) criteria, with low risk of suicide, i.e. no thoughts of death and no previous attempts^45^;
- absence of cognitive impairment and substance abuse;
- either in a hypomanic phase or a moderately depressive phase period.

Note that the mixed state was not considered as a possible status in the first study period. Moreover, patients with Major Depressive Disorder (MDD) were not included in the sample.

All the subjects signed the informed consent for the PSYCHE project that was approved by the Ethical Committee of Strasbourg. All experimental procedures and analyses were carried out in accordance with such approved guidelines and regulations.

Eight patients were selected for this study and their demographic information is reported in Table 1.

**Table 1:** Patients demographic and clinical information, including age, gender, illness duration, bipolar disorder (BD) subtype, and the number of day and night trials.

Patient	Age	Gender	Illness duration	BD subtype	Num. day trials	Num. night trials
Pz01	39	M	1.0 yr	II	6	2
Pz02	51	M	14.5 yrs	I	5	3
Pz03	39	M	14.8 yrs	I	4	2
Pz04	27	M	10.2 yrs	I	6	2
Pz05	54	F	11.1 yrs	II	3	2
Pz06	33	M	14.6 yrs	I	1	1
Pz07	37	F	12.8 yrs	I	6	3
Pz08	29	M	5.9 yrs	I	5	1

Diagnoses were performed by board-certified psychiatrists and clinical psychologists. The protocol planned a study entry visit when the patient was experiencing a depressive or hypomaniac state. The initial diagnosis of the current bipolar episode was determined by clinicians according to DSM-IV-TR criteria. Patients were studied with an average frequency of 2–3 times a month. Patients were evaluated and monitored from the day of hospital admission until remission, i.e., until they reached euthymic state. In this study no more than six evaluations per patient were performed. The evolution of a mood state to another, such as transition from hypomania to depressive state or euthymic state, was assessed using scores from quantitative psychopathological rating scales. This was a purely clinical evaluation that disregarded any physiological/biochemical reference analysis. The mood label associated with each patient evaluation was assigned independently with respect to the previous ones. The euthymic state was defined as having a score below threshold on a quantitative psychopathological rating scale: for depressive symptoms, below 8 on the 16-item Quick Inventory of Depressive Symptomatology, Clinician-Rated QIDS-C16, and for manic symptoms below 6 on the Young Mania rating scale, YMRS^29,46^. The same thresholds were also used to define a change in mood state. During the study, treatment choice remained at the discretion of the clinician as well as the change of treatment in case of lack of response.

The set of patient acquisitions was comprised of 16 night recordings (6 in depressive state, 5 with euthymia and 5 with hypomanic mood) and 36 day recordings (13 related to depressive state, 9 to euthymic and 14 to hypomanic state).

We also analyzed the heartbeat dynamics of a group of 11 healthy female subjects.

Subjects in the control group, in the age range of 18–45, were not affected by:

- any past or current psychiatric disorders as evidenced in the Mini International Neuropsychiatric Interview (MINI) during a psychological examination and an assessment with the QIDS (exclusion if the score was higher than 6);
- personal history or family history of psychiatric disorders;
- any physical disorder;
- chronic medication.

The set of healthy subject acquisitions was comprised of 11 daytime and 11 nighttime recordings.

### MultiScale entropy analysis

The MSE algorithm is based on the application of the SampEn technique to coarse-grained time series constructed from the original signal by averaging the data points within non-overlapping windows of increasing length, *τ*. Given a time series {*x*_1_, …, *x*_*i*_, …, *x*_*N*_} and a scale factor *τ*, each element of a coarse-grained series {*y* ^(*τ*)^} is calculated using the equation

1 $${y}_{j}^{(\tau )}=\frac{1}{\tau }\sum _{i=(j-\mathrm{1)}\tau +1}^{j\tau }{x}_{i},\,1\le j\le N/\tau $$

where the length of each coarse-grained time series is equal to the length of the original time series divided by *τ*. The second step consists of the computation of SampEn algorithm in these series^37,47^.

This technique involves two parameters: *m*, a positive integer, which is the size of the compared patterns, and *r*, a positive real number, which is multiplied by the standard deviation of the series and represents the margin of tolerance in the comparison.

Starting from the vectors *x*(1), *x*(2), …, *x*(*N* − *m* + 1) in ${{\mathbb{R}}}^{m}$ defined by *x*(*i*) = [*u*(*i*), *u*(*i* + 1), ..., *u*(*i* + *m* − 1)], the distance between two vectors *x*_1_ and *x*_*j*_ is calculated according to the definition given by Takens in his studies on high-dimensional deterministic systems^48,49^:

2 $$d[x(i),x(j)]=ma{x}_{k=\mathrm{1,2,...,}m}|u(i+k-\mathrm{1)}-u(j+k-\mathrm{1)|}$$

For each *i*, with 1 ≤ *i* ≤ *N* − *m* + 1, the parameter ${C}_{i}^{m}(r)$ is evaluated using the following equation:

3 $${C}_{i}^{m}(r)=\frac{{\rm{Number}}\,{\rm{of}}\,{\rm{j}}\,{\rm{such}}\,{\rm{that}}\,(d[x(i),x(j)]\le r)}{N-m+1}$$

where *i* ≠ *j*.

SampEn is calculated with the expression

4 $${\rm{SampEn}}({\rm{m}},{\rm{r}},{\rm{N}})=-\mathrm{ln}\,\frac{{C}^{m+1}}{{C}^{m}}$$

where *C* ^*m*^ is given by:

5 $${C}^{m}(r)=\frac{\sum _{i=1}^{N-m+1}\,\mathrm{log}\,{C}_{i}^{m}(r)}{N-m+1}$$

Results from the MSE analysis are reported plotting the curve where each value of SampEn, calculated over 20 scales, is represented as a function of the related scale factor *τ*. The time scale range, from 1 to 20, is the standard suggested in the literature as related to MSE analysis^33,35,43^. It has been suggested in^33,35^, considering the 20^th^ time scale as the asymptotic resampling value to study heartbeat multiscale fluctuations. Moreover, the same range was used to discern depressed from non-depressed subjects using nighttime ECG recordings^43^.

Regarding the choice of parameters *m* and *r*, in this study we followed the procedure suggested in^50^. Of note, previous studies investigating different methods for the calculation of parameter *r* demonstrated that this procedure was the most effective and appropriate for heartbeat complexity analysis in bipolar disorder^28^.

More specifically, this calculation foresaw searching for the value of *r* which maximized the calculation of *ApEn* in the range 0.01 ≤ *r* ≤ 1.2. The highest value *ApEn*(*r*_*k*_) was interpolated with the preceding and the following values, *ApEn*(*r*_*k*−1_) and *ApEn*(*r*_*k*+1_), with a parabola and the position of the vertex of the parabola gave the value of *r*_*max*_. Therefore, *ApEn*(*r*_*max*_) quantified the highest information difference between vectors of dimensions *m* and *m* + 1. The value of the pattern dimension, *m*, was set at 2, according to the most common value used in the literature.

Furthermore, to summarize the MSE results over all scales, we evaluated the so-called Complexity Index (CI) by calculating the area under the MSE curve^27,43^. This procedure was performed using numerical integration via the trapezoidal method. The area under curve was calculated for short scales, from 1 to 8, and higher time scales, from 1 to 20. The eighth scale was considered as the threshold for short time-scales according to a previous study dealing with MSE application for the assessment of mental disorders^43^. This cutoff value limits the scales expressing the dynamics in the high-frequency band. The traditional cutoff used in HRV frequency analysis is indeed 0.15 Hz, so given that the mean of RR interval lengths in^43^ was 0.81 seconds, we considered scales from 1 to 8 as short time-scales. The role of sympathetic and parasympathetic control of heart rate complexity upon different time-scales has been recently investigated in rats^51,52^. These studies demonstrated that short scales in the MSE curve express the effect of the vagal control of the heart and suggest that regulatory mechanisms other than baroreflex might contribute to nonlinear HRV features observed at short time scales. The sympathetic control keeps the unpredictability of RR fluctuations at higher scales, and a high sympathetic tone contributes to limiting nonlinear HRV components via a vagal withdrawal at short time scales. Nevertheless, the combined blockade of cardiac autonomic receptors produces an increase of irregularity at short scales and highlights a higher robustness of the MSE curve profile rather than the single entropy value to provide an index of heart rate dynamics^51^.

### Statistical Analysis

For each index, Kruskal-Wallis non-parametric tests were employed to investigate significant differences among healthy control states and pathological mood states including euthymia, depression, and hypomania. In this case, the null hypothesis was associated with having no differences in medians among samples. Mann-Whitney non-parametric U-tests were used to compare data of two different groups, for example, euthymia versus hypomania, through *post*-*hoc* statistical analysis, using Bonferroni’s correction. Of note, the use of such non-parametric tests was justified by having non-gaussian distributions associated with samples, tested with Shapiro-Wilk procedure^53^.

Statistical analysis was performed separately on daytime and nighttime segments to investigate differences among mood states. Moreover, differences between daytime and nighttime segments for each patient/mood state were also studied.

## Results

For each acquisition, we analyzed the longest artifact-free segment and expressed all the group-wise results as median and median absolute deviation (MAD).

### Heartbeat Complexity modulation in Healthy Subjects and Bipolar patients during daytime and nighttime

**Figure 1:**
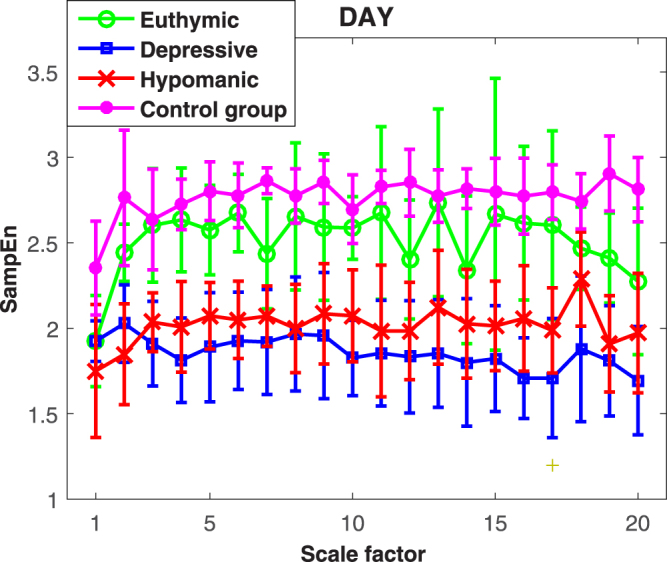
MSE of heartbeat dynamics in healthy control subjects and bipolar patients during daytime monitoring. Values are expressed as *median* ± *MAD*.

**Figure 2:**
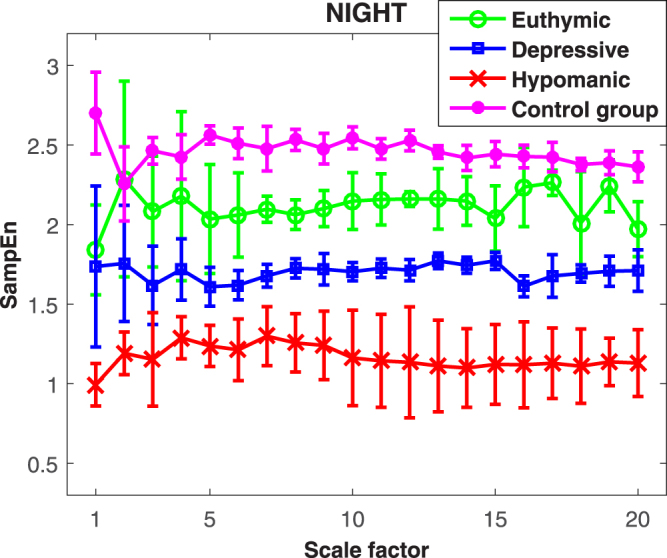
MSE of heartbeat dynamics in healthy control subjects and bipolar patients during nighttime monitoring. Values are expressed as *median* ± *MAD*.

MSE values from healthy subjects and bipolar patients are shown in Figs 1 and 2 for daytime and nighttime, respectively. In both figures, it is possible to note that a higher irregularity level is associated with the healthy state with respect to the pathological ones.

Specifically concerning daytime monitoring, overall comparable entropy values were found for the healthy and the euthymic states, whereas a significant lower irregularity level was associated with the hypomanic and depressive states. The Kruskal-Wallis test revealed significant statistical differences over all scales with *p* < 0.001, with the exception of the first scale which was associated with *p* < 0.03. *Post*–*hoc* Mann-Whitney tests did not reveal significant differences between the healthy and euthymic states (*p* > 0.05). On the other hand, significant differences between healthy and depressive states at all scales (*p* < 0.05) and between healthy and hypomanic states at scales from 2 to 20 (*p* < 0.01) were found. Euthymic states were significantly different from depressive state at scales {2–4, 6, 7, 11–14, 16–19} (*p* < 0.05) and from hypomanic states at scales {2–4, 6, 17} (*p* < 0.05). Entropy values from depressive and hypomanic states did not show significant differences at all scales.

Concerning nighttime monitoring, the Kruskal-Wallis test revealed significant differences among the four states with *p* < 0.01 at all scales. *Post*–*hoc* Mann-Whitney tests did not reveal significant differences between the healthy and euthymic states (*p* > 0.05). Significant differences between healthy and depressive states at 9 scales {1–4, 6–8, 12, 13} (*p* < 0.01), and between healthy and hypomanic states at all scales (*p* < 0.01) were also found. Euthymic states were not significantly different from depressive states, and from hypomanic states at scales {1, 6–20} (*p* > 0.05). Entropy values from depressive and hypomanic states showed significant differences at scale 1 (*p* < 0.05).

#### Complexity Index Analysis

**Figure 3:**
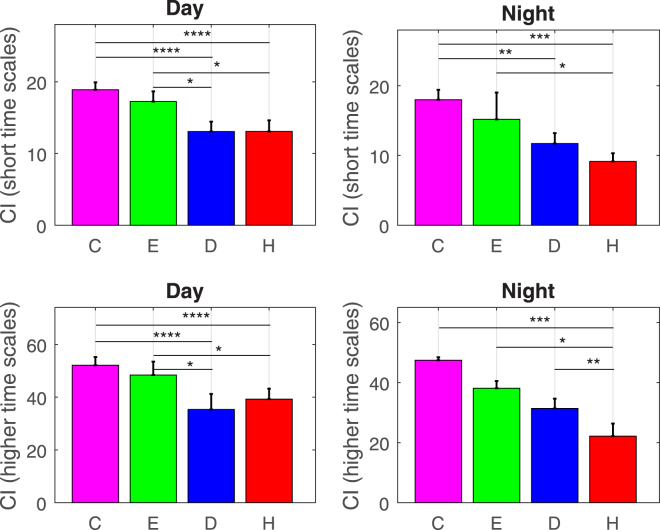
Bar graphs of complexity index analysis on short (calculated using time-scales from 1 to 8, top panel), and higher (calculated using time-scales from 1 to 20, bottom panel) time scales. The results of statistical tests are expressed with the symbols: ****p < 0.001, ***p < 0.01, **p < 0.03, *p < 0.05. C = control group, E = euthymic, D = depressive, H = hypomanic.

To summarize the results achieved throughout all MSE scales and to provide a meaningful index for pathological mood state assessment using cardiovascular dynamics exclusively, a complexity index (CI) analysis was carried out. Results are shown in Table 2, and summarized in Fig. 3.

**Table 2:** CI calculated on the MSE graphs for diurnal and nocturnal periods, values are *median* ± *MAD*.

	Short Time Scales	Higher time scales
**Diurnal acquisitions**		
Control group	18.89 ± 1.03	52.11 ± 3.10
Euthymic	17.25 ± 1.42	48.40 ± 5.05
Depressive	13.06 ± 1.38	35.35 ± 5.86
Hypomanic	13.08 ± 1.55	39.25 ± 3.97
p-value	8.30*e* ^−6^	5.51*e* ^−6^
**Nocturnal acquisitions**		
Control group	17.99 ± 1.42	47.43 ± 1.01
Euthymic	15.18 ± 3.85	38.11 ± 2.40
Depressive	11.71 ± 1.50	31.39 ± 3.26
Hypomanic	9.14 ± 1.18	22.17 ± 4.17
p-value	6.99*e* ^−4^	8.81*e* ^−4^

Specifically concerning daytime monitoring, we applied Kruskal-Wallis tests on CI values calculated over the MSE scales among the four samples. We found significant differences considering both the short-time and-higher time scales (*p* < 10^−5^). Healthy state was not significantly different from the euthymic state. Healthy state was significantly different from the depressive and hypomanic states with *p* < 0.001 for short and higher time scales. Euthymic state was significantly different from the depressive and hypomanic states with *p* < 0.05 for short and higher time scales. Depressive and hypomanic states did not show significant CI-related differences.

Concerning nighttime monitoring, Kruskal-Wallis tests revealed significant differences among all states on CI short scales and higher scales (*p* < 0.001). Healthy state was significantly different from the depressive states with *p* < 0.03 for short time scales, and significantly different from the hypomanic states with *p* < 0.01 for short and higher time scales. However, healthy state was not significantly different from the euthymic state. Euthymic state was significantly different from the hypomanic states with *p* < 0.05 for short and higher time scales, whereas depressive and hypomanic states showed significant differences with *p* < 0.03, for higher scales.

### Heartbeat Complexity modulation between daytime and nighttime

We observed that heartbeat dynamics became more regular during the nighttime with respect to daytime. Specifically, a decrease in the entropy values during the manic and euthymic states, with a reduction of more than 40% in the higher time scales, compared to decrease of less than 10% in the control group were observed. Furthermore, Mann-Whitney non-parametric statistical tests revealed significant differences between daytime and nighttime periods for all mood states with p < 0.05. In Table 3 results from the statistical analysis related to CI values are shown.

**Table 3:** p-values from the Mann-Whitney test comparing CI values between daytime and nighttime periods, over short and higher time scales.

	Control group	Euthymic	Depressive	Hypomanic
CI (short time scales)	0.030 (d)	0.001 (d)	0.017 (d)	0.019 (d)
CI (long time scales)	0.003 (d)	0.001 (d)	0.029 (d)	0.014 (d)

Symbols (d) and (n) indicate that SampEn value increased during the day or during the night, respectively. Bold indicates p-values lower than 0.05.

Likewise, significant differences were found investigating daytime and nighttime heartbeat dynamics for each mood state across all scales (see Table 4). p-values from Mann-Whitney non-parametric statistical tests were below the threshold of 0.05 in almost all cases, with the exception of some short time-scales for the control group and some higher time-scales for the depressive group.

**Table 4:** p-values from the Mann-Whitney test comparing SampEn estimates between daytime and nighttime periods over all the 20 time scales.

	Controls	Euthymic	Depressive	Hypomanic
MSE (scale 1)	**0.049 (n)**	**0.012 (d)**	**0.001 (d)**	**0.0003 (d)**
MSE (scale 2)	**0.042 (d)**	**0.001 (d)**	**0.029 (d)**	**0.002 (d)**
MSE (scale 3)	0.694 (d)	**0.001 (d)**	**0.007 (d)**	**0.034 (d)**
MSE (scale 4)	**0.013 (d)**	**0.001 (d)**	**0.017 (d)**	**0.026 (d)**
MSE (scale 5)	0.149 (d)	**0.001 (d)**	**0.012 (d)**	**0.034 (d)**
MSE (scale 6)	**0.009 (d)**	**0.001 (d)**	**0.012 (d)**	**0.019 (d)**
MSE (scale 7)	**0.001 (d)**	**0.001 (d)**	**0.017 (d)**	**0.019 (d)**
MSE (scale 8)	**0.042 (d)**	**0.001 (d)**	**0.012 (d)**	**0.019 (d)**
MSE (scale 9)	**0.0003 (d)**	**0.029 (d)**	**0.022 (d)**	**0.014 (d)**
MSE (scale 10)	**0.018 (d)**	**0.002 (d)**	**0.029 (d)**	**0.019 (d)**
MSE (scale 11)	**0.001 (d)**	**0.001 (d)**	0.072 (d)	**0.005 (d)**
MSE (scale 12)	**0.018 (d)**	**0.001 (d)**	0.106 (d)	**0.005 (d)**
MSE (scale 13)	**0.005 (d)**	**0.001 (d)**	0.179 (d)	**0.007 (d)**
MSE (scale 14)	**0.001 (d)**	**0.001 (d)**	0.282 (d)	**0.005 (d)**
MSE (scale 15)	**0.001 (d)**	**0.004 (d)**	**0.046 (d)**	**0.005 (d)**
MSE (scale 16)	**0.001 (d)**	**0.004 (d)**	0.058 (d)	**0.005 (d)**
MSE (scale 17)	**0.003 (d)**	**0.001 (d)**	0.179 (d)	**0.005 (d)**
MSE (scale 18)	**0.0001 (d)**	**0.004 (d)**	0.152 (d)	**0.002 (d)**
MSE (scale 19)	**0.001 (d)**	**0.001 (d)**	0.106 (d)	**0.005 (d)**
MSE (scale 20)	**0.001 (d)**	**0.004 (d)**	0.179 (d)	**0.003 (d)**

(d)Indicates that SampEn value increased during the day, (n) points out an increase of SampEn during the night. Bold indicates p-values lower than 0.05.

## Discussion

In this study, we investigated how heartbeat complex dynamics is affected by pathological mood states in bipolar disorder during the day and night.

Previous studies already proposed the use of MSE methods to examine differences between depressive state patients and healthy subjects^42,43,54^, with a significantly reduced heartbeat irregularity associated with pathological states. Of note, the decrease of irregularity level has often been associated with advanced aging and other diseases such as congestive heart failure^55–58^. We built on these studies to further investigate heartbeat complexity modulation among pathological mood states in bipolar disorder such as depression, hypomania and euthymia, also comparing MSE-related indices with data from a healthy control group.

Our findings confirm the results of Leistedt *et al*., who compared patients affected by major depression and healthy subjects during the night^43^. We demonstrated that bipolar depressive mood is also associated with a decrease in the HRV irregularity with respect to the euthymic state, being statistically significant across the short-time scales. Healthy subjects showed higher entropy values with respect to all pathological mood states at the higher scales. This is in line with outcomes highlighted by Costa *et al*. who analyzed the MSE curves of healthy young and elderly subjects, using nighttime recordings^35^. Also in this case, the difference between the two groups was significant only over short scales, which are related to the high-frequency modulation of the cardiac rhythm. Their results showed lower values of entropy in the elderly group and were consistent with the loss of parasympathetic modulation with age^59^. At a speculation level, trends we reported for depressive state subjects support the link between aging and depression, already known in the literature^60^. We found a reduced heartbeat irregularity not only between healthy and depressive states, but also between depression and hypomania when compared with euthymic and healthy states. Such a cardiovascular complexity modulation was more evident analyzing nighttime recordings. Euthymic and depressed patients exhibited values of entropy which were statistically different only during the day. During the nighttime period the signals acquired in hypomanic patients were demonstrated to be distinguishable from the euthymic subjects both for short and higher scales. Depression and euthymia were not different, according to the statistical tests, when the short scales were analyzed during the night. At a speculation level, this outcome can be explained with some sleep-related functioning in bipolar patients^61,62^. Particularly, a previous study demonstrated that 70% of the euthymic patients with bipolar disorder exhibited a clinically-significant sleep disturbance such as insomnia^61^. Insomnia and hypersomnia are also the most typical sleep-related disorders associated to bipolar depression^63^.

Changes in HRV complexity between day and night have already been reported in the literature. Exemplarily, Costa *et al*. studied young healthy subjects, elderly healthy subjects and heart failure subjects^35^. In all these groups they found statistically significant differences between day and night, using the MSE algorithm. In the healthy young subjects group the entropy decreased during the night, whereas in elderly and pathological subjects an increase of complexity was found.

Our results are in agreement with lower entropy observed during the night with respect to day across all mood states. Although evidence in animal studies has suggested that *α*-adrenoceptors, the cholinergic system, as well as adenosine 3′,5′-cyclic monophosphate are responsible for complex cardiovascular fluctuations^24,64,65^, the actual correlates of autonomic activity on complex cardiac control are still unknown.

Our study does present some limitations. The first is that we considered female healthy control subjects only. This surely limits the generalization of our results, which might be different when considering a representative sample of the healthy population including both male and female subjects. Inclusion criteria for healthy subjects foresaw a slightly different age range (18–45) than patients (27–54) and, although minimally, this might slightly bias the MSE results.

Moreover, although a complexity modulation between healthy subjects and euthymic state occured, there was no statistical significance associated with this comparison after correction for multiple comparison. This could be due to the low number of subjects involved in the study, as well as to the very high inter-subject variability associated with bipolar disorder. Importantly, to improve on the homogeneity of the pathological group, we reduced the number of patients according with illness duration and type of disease, removing Pz01 and Pz08 data to reduce illness duration variability, or removing Pz01 and Pz05 data for a bipolar disease type I sample. We found that the control group showed a significantly higher complexity than euthymic group during the night (see Supplementary Material for further details). Future endeavours comparing different types or clinical history of bipolar disease are thus needed, maybe investigating whether baseline HRV complexity could be an indicator of bipolar disease severity.

Daytime and nighttime evaluations were based on absolute timing considerations only. No quantitative measures were taken into account to evaluate REM latency and sleep stages. Furthermore, some of the patient recordings took place in a research center 80 kilometers from the clinical center and home. They had to be autonomous to travel on their own to the research center. For security reasons, we excluded patients at risk of suicide attempts, such as personal antecedents or current ideation, and with severe depression or full mania. A substantial part of patients with MDD is actually a candidate of bipolar disorder, i.e. ‘Latent Bipolar Disorder’, but were not included in the sample. Finally, while summarizing the MSE results through CI allows for a more concise description of the results, this might be associated with the loss of some information. We also remark that one of the experimental results shown in this article (MSE in bipolar patients during nighttime only) was already reported in our previous methodological endeavour^28^, and has been included here for comparative purposes and completeness of description exclusively.

Despite these limitations, although preliminary, our findings surely suggest that bipolar disorder is associated with an enhanced sleep-related dysregulation of the ANS activity and the use of heartbeat complex dynamics as viable markers of pathological mental conditions. This conclusion is supported by previous evidence suggesting nonlinear analysis of physiological signals to support care in mental health^66–68^, thus possibly overcoming the use of scores from structured tests only.

Indeed, there is compelling evidence of disrupted circadian rhythms in individuals with mood disorders^63,69,70^. In particular, sleep disturbance and circadian dysregulation are critical pathophysiological elements in bipolar disorder^69^, and our findings could help answer the many questions related to their underpinning mechanisms. Moreover, a bidirectional relationship between daytime affect regulation and nighttime sleep has been recently recognized, also because neurotransmitters in brain regions implicated in mood regulation exhibit circadian rhythms^63,69,70^. A previous study also suggested that the multiscale influences of the suprachiasmatic nucleus on heart rate fluctuations in rats cannot be explained by a simple pacemaker of 24 hours rhythmicity^71^. To this extent, future endeavours can be directed to the study of heartbeat dynamics in bipolar subjects over 24 hours in order to check the intrinsic circadian variation of HRV among different mood states.

Our results are also in line with the theory hypothesizing that psychiatric diseases are multi-system disorders, associated with a degeneration of the physiological systems and consequent cardiovascular diseases, cancer and accelerated aging^72^. It is thought that cardiovascular irregularity arises from the interaction of several neuronal signaling and multi-feedback operating over a wide range of temporal and spatial scales, possibly associated with the neuroendocrine regulation. A previous study, in fact, underlined the role of *β*-adrenoceptors and *α*-adrenoceptors as factors which influence the nonlinear and fractal dynamics of heartbeat^64^.

Future endeavors will be directed to the exploitation of these results in conjunction with other significant changes associated with pathological mood swings in bipolar disorder. Moreover, since recent studies have proposed alternative approaches to measure the irregularity of short-term physiological series, for instance distribution entropy (DistEn)^73^, future work will focus on the application of these promising algorithms for entropy calculation in the field of mood disorders and emotion recognition.

## Electronic supplementary material

Supplementary Material
