# Supplementary material for: Heartbeat Complexity Modulation in Bipolar Disorder during Daytime and Nighttime
Source: Sci Rep. 2017 Dec 20;7:17920. doi: 10.1038/s41598-017-18036-z (PMC5738374; doi:10.1038/s41598-017-18036-z)
Supplement: Supplementary file 1 — Supplementary Material [file 41598_2017_18036_MOESM1_ESM.pdf]

Supplementary Material: Heartbeat Complexity Modulation in Bipolar Disorder during Daytime and Nighttime

Mimma Nardelli[1,*], Antonio Lanata[1], Gilles Bertschy[2], Enzo Pasquale Scilingo[1], Gaetano Valenza[1]

[1]Computational Physiology and Biomedical Instruments group, Department of Information Engineering & Bioengineering and Robotics Research Centre E. Piaggio, School of Engineering, University of Pisa, Pisa, Italy

[2]Department of Psychiatry and Mental Health, Strasbourg University Hospital, INSERM U1114, University of Strasbourg - F-67000 Strasbourg, France

[*]Corresponding Author

Starting from the demographic and clinical information of bipolar patients involved in this study, we here investigate two supplementary cases. Specifically, we calculated the values of complexity index (CI) considering the following sub-datasets of patients:
- case 1: original dataset without Pz01 and Pz08;
- case 2: original dataset without Pz01 and Pz05.

These additional analyses have been performed in order to explore if the illness duration of Pz01 and Pz08 (case 1), or the different type of disease of Pz01 and Pz05 (case 2), may affect the observed changes across mood states.
Kruskal-Wallis and Mann-Whitney statistical tests have been applied to discern the four groups statistically. A *post-hoc* analysis was performed on the results of Mann-Whitney test using Bonferroni's correction. Nevertheless, it is important to note that the non-inclusion of data from patients Pz01-Pz08 in case 1 and non-inclusion of data from Pz01-Pz05 in case 2 resulted in a reduction of 14 and 13 acquisitions/observations (considering both nighttime and daytime), respectively, therefore significantly decreasing the statistical power associated with the analysis.

Case 1: Illness duration

In Figure **??** the results of statistical analysis on control group and patients in case 1 are shown. The results of Kruskal-Wallis test were statistically significant during the day ($p = 0.00009$) and the night ($p = 0.0002$) for short scales, and also during daytime ($p = 0.00007$) and nighttime ($p = 0.0002$) for higher scales. By removing Pz01 and Pz08 from the dataset, the three pathological states do not result to be statistically different, whereas the control group is still significantly different from pathology.

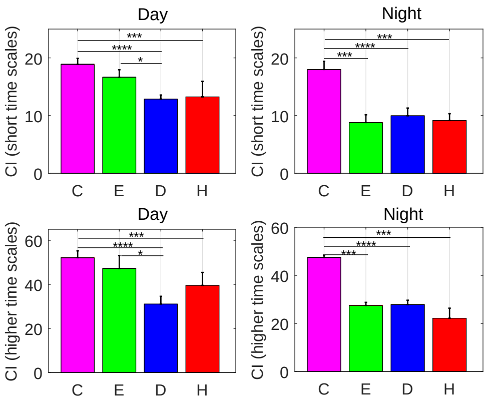

Figure i: Bar graphs of complexity index analysis on short (top panel) and higher (bottom panel) time scales, without considering Pz01 and Pz08. The results of statistical tests are expressed with the symbols: $**** \equiv p < 0.001$, $*** \equiv p < 0.01$, $** \equiv p < 0.03$, $* \equiv p < 0.05$. C= control group, E= euthymic, D= depressive , H= hypomanic.

Case 2: Analysis on Bipolar Disorder type I

In Figure **??** the results of statistical analysis on healthy subjects and patients affected by only Bip. type I are shown. The results of Kruskal-Wallis test were significant during daytime ($p = 0.00008$) and nighttime ($p = 0.0002$) for short scales, and during day-time ($p = 0.00005$) and night-time ($p = 0.0002$) for higher scales.

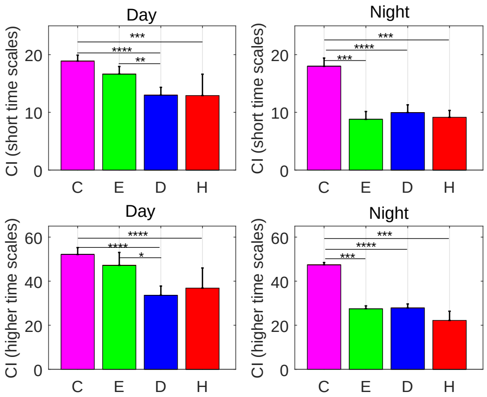

Figure ii: Bar graphs of complexity index analysis on short (top panel) and higher (bottom panel) time scales, without considering Pz01 and Pz05. The results of statistical tests are expressed with the symbols: $**** \equiv p < 0.001$, $*** \equiv p < 0.01$, $** \equiv p < 0.03$, $* \equiv p < 0.05$. C= control group, E= euthymic, D= depressive , H= hypomanic.
